# Phylogenetic analysis of the non-structural (NS) gene of influenza A viruses isolated from mallards in Northern Europe in 2005

**DOI:** 10.1186/1743-422X-5-147

**Published:** 2008-12-12

**Authors:** Siamak Zohari, Péter Gyarmati, Anneli Ejdersund, Ulla Berglöf, Peter Thorén, Maria Ehrenberg, György Czifra, Sándor Belák, Jonas Waldenström, Björn Olsen, Mikael Berg

**Affiliations:** 1 Joint Research and Development Unit for Virology, Immunobiology, and Parasitology, of the National Veterinary Institute (SVA) and Swedish University of Agricultural Sciences (SLU), and Department of Biomedical Sciences and Public Health, Section of Parasitology and Virology, SLU, Ulls väg 2B, SE-751 89 Uppsala, Sweden; 2 Unit for Virology, Immunobiology, and Parasitology, SVA, Ulls väg 2B, SE-751 89 Uppsala, Sweden; 3 Unit for chemistry, environment and feed safety of National Veterinary Institute (SVA) Ulls väg 2B, SE 751 89 Uppsala, Sweden; 4 Department of Medical Sciences, Section of Infectious Diseases, Uppsala University Hospital, SE 751 85 Uppsala, Sweden; 5 Section for Zoonotic Ecology and Epidemiology, Kalmar University, SE-321 85 Kalmar, Sweden

## Abstract

**Background:**

Although the important role of the non-structural 1 (*NS*) gene of influenza A in virulence of the virus is well established, our knowledge about the extent of variation in the *NS *gene pool of influenza A viruses in their natural reservoirs in Europe is incomplete. In this study we determined the subtypes and prevalence of influenza A viruses present in mallards in Northern Europe and further analysed the *NS *gene of these isolates in order to obtain a more detailed knowledge about the genetic variation of *NS *gene of influenza A virus in their natural hosts.

**Results:**

A total number of 45 influenza A viruses of different subtypes were studied. Eleven haemagglutinin- and nine neuraminidase subtypes in twelve combinations were found among the isolated viruses. Each NS gene reported here consisted of 890 nucleotides; there were no deletions or insertions. Phylogenetic analysis clearly shows that two distinct gene pools, corresponding to both NS allele A and B, were present at the same time in the same geographic location in the mallard populations in Northern Europe. A comparison of nucleotide sequences of isolated viruses revealed a substantial number of silent mutations, which results in high degree of homology in amino acid sequences. The degree of variation within the alleles is very low. In our study allele A viruses displays a maximum of 5% amino acid divergence while allele B viruses display only 2% amino acid divergence. All the viruses isolated from mallards in Northern Europe possessed the typical avian ESEV amino acid sequence at the C-terminal end of the NS1 protein.

**Conclusion:**

Our finding indicates the existence of a large reservoir of different influenza A viruses in mallards population in Northern Europe. Although our phylogenetic analysis clearly shows that two distinct gene pools, corresponding to both *NS *allele A and B, were present in the mallards populations in Northern Europe, allele B viruses appear to be less common in natural host species than allele A, comprising only about 13% of the isolates sequenced in this study.

## Background

Several viral gene products of influenza A virus are known to contribute to the host range restriction and virulence of the virus. The viral polymerase protein 2 (PB2) with its amino acid at position 627 influences the ability of the virus to replicate in human or mouse cells [1]. The receptor binding efficiency and high cleavability of the haemagglutinin (HA) glycoprotein can influence viral entry and lethal out come of infection [2]. The non-structural protein 1 (NS1) which is a multi-functional protein, plays a crucial role in viral virulence by countering cellular antiviral activities [3] and contributes to virus replication by participating in multiple protein-RNA and protein-protein interaction.

The *NS *gene of influenza A viruses encodes an mRNA transcript that is alternatively spliced to express two proteins [4]. Translation of the unspliced mRNA encodes a 26-kDa NS1 protein which shares the same ten amino acids from the initiation codon at the N-terminal of the protein with a 14-kDa nuclear export protein (NEP, formerly called NS2) which is translated from spliced mRNA [5]. Depending on virus strain NS1 consists of 124–237 amino acids in length and is expressed exclusively in infected cells.

The NS1 protein contains two functional domains: the N-terminal RNA-binding domain (residues 1–73) and the C-terminal effector domain (residues 73–237) [6].

It has been suggested that the N-terminal RNA binding domain of NS1 protein has regulatory activities that are important to prevent interferon mediated antiviral responses. Binding of NS1 protein to both single- and double-stranded RNA might: (a) inhibit activation of interferon induced protein kinase PKR [7], (b) prevent activation of the 2'–5'oligoadenylate synthetase, which is essential for activation of ribonuclease L (RNase L) system [8], (c) inhibit the activation of IRF-3 and NF-κB, key regulators of IFN α and β gene expression, by interfering with the retinoic acid-inducible gene I (RIG-I) [9-11] and (d) suppression of RNA interfering system, by binding to small interfering RNAs [12,13]. Earlier studies have indicated the existence of important amino acid sequence motifs for the function of NS1 protein. Analysis implies that amino acids at the N-terminal RNA-binding domain of NS1 are implicated in this function. The arginine at position 38 and the lysine at position 41 contribute to this interaction [10]. The N-terminal residues 81–113 of NS1 protein can also bind to eukaryotic translation initiation factor 4GI (eIF4GI), the large subunit of the cap-binding complex eIF4F [14]. By doing so, NS1 protein recruits eIF4F to the 5' un-translational region of viral mRNA and activates translation of viral mRNA.

The effector domain of NS1 protein has been associated with regulation of gene expression of the infected cell [15]. It has been shown that the effector domain of NS1 protein: (a) inhibit 3'-end processing of cellular pre-mRNA by specifically interaction with the 30 kDa subunit of the cleavage and polyadenylation specific factor (CPSF) [16-18]. This function mediated by two distinct domains; one located around residue 186 [18] and the other one around residue 103 and 106 [19], (b) prevent transport of cellular mRNA to cytoplasm by interaction with poly (A) – binding protein II (PABII) [20]. Amino acids 215 to 237 have been identified as the binding site for PABII [18].

The NEP consists of 121 amino acids [21] which in association with the matrix protein 1 (M1) interacts with cellular export factor (CEF1) and mediate the nuclear export of viral ribonucleoprotein complexes [22] by connecting the cellular export machinery with vRNPs [23].

Our knowledge about the *NS *gene pool of influenza A viruses in their natural reservoirs in Europe is incomplete. Limited information on the prevalence of influenza A viruses in wild birds in Europe has been provided in recent years indicating Mallards (*Anas platyrhynchos*) as an essential factor of the ecology of influenza A viruses because of a particularly wide variety of subtypes isolated from these birds [24-28]. Therefore, in this study we analysed in detail the *NS *gene sequences of 45 influenza A viruses, isolated from mallards at the major flyway of the Western Eurasian mallard population in 2005, in order to gain more detailed knowledge about the genetic variation of influenza A viruses in their natural hosts.

## Results and discussion

### Avian influenza Prevalence

**Figure 1:**
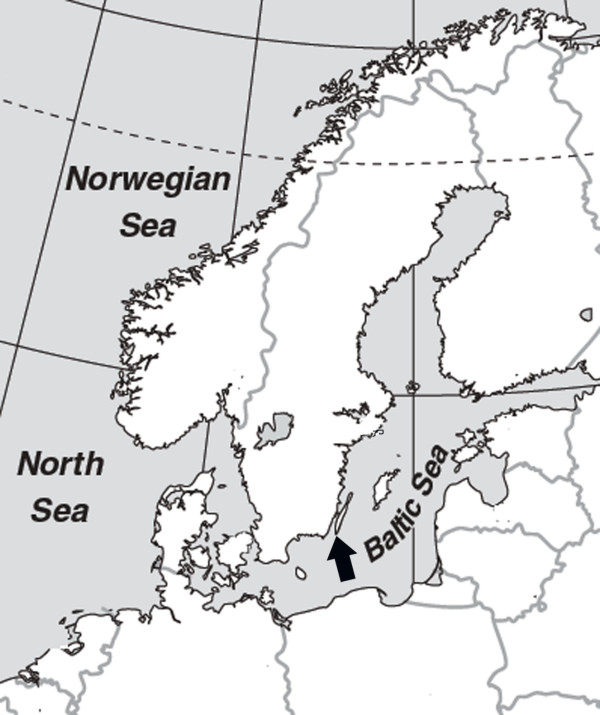
The sample location at Ottenby bird Observatory (56°12' N, 16°24' E) on a major European flyway, on Baltic island of Öland at southeast coast of Sweden indicated by a black arrow.

**Figure 2:**
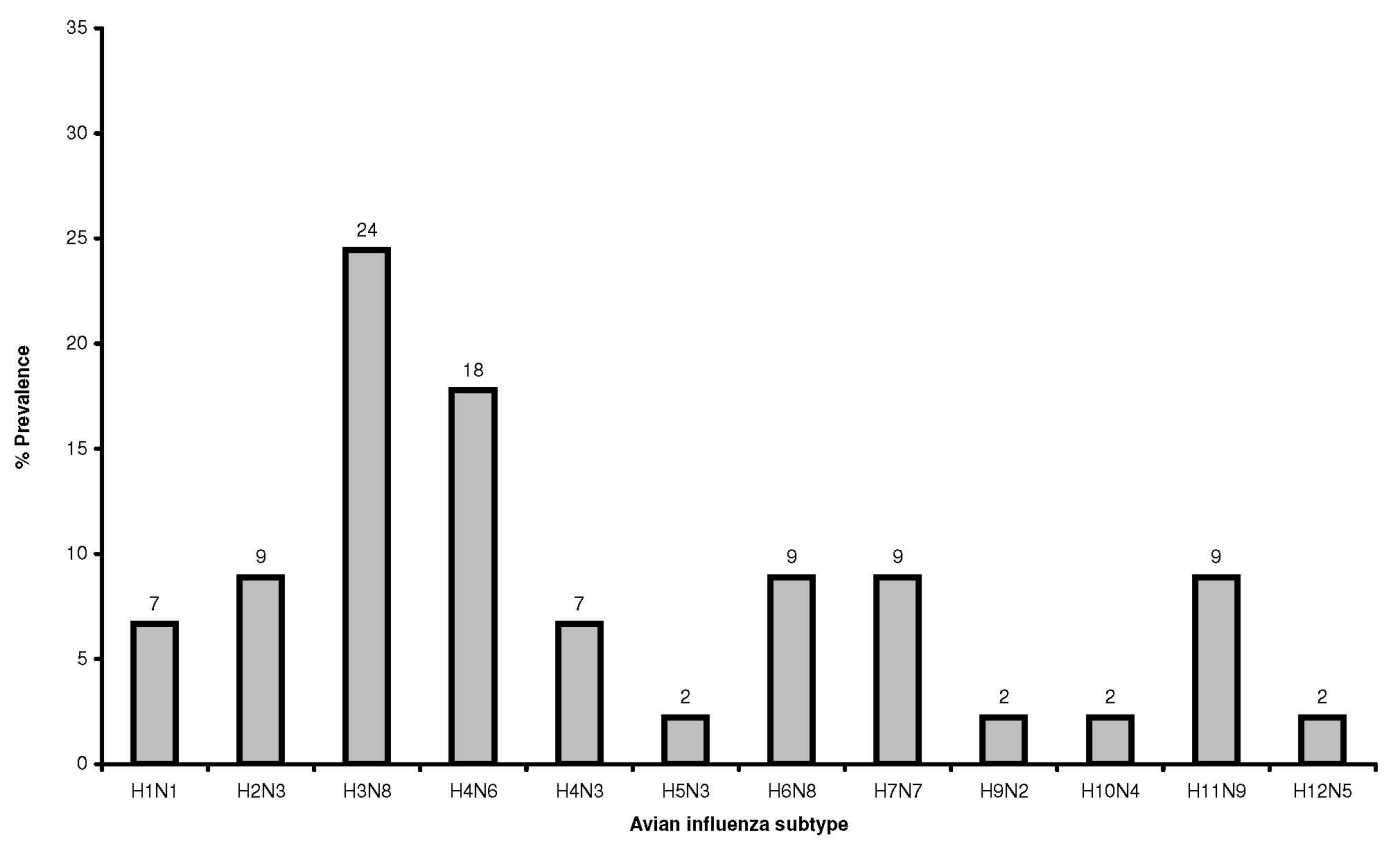
Prevalence of each influenza A virus subtype isolated from mallards in Northern Europe in 2005.

Samples from seven hundred and eighty one mallards (*Anas platyrhynchos*) were collected in the frame of a surveillance program, organized by the Swedish Board of Agriculture (Figure 1). Birds were caught from October until the autumn migrations were ended in late December. The matrix real-time reverse transcriptase polymerase chain reaction (rRT-PCR) screening showed that about 24% of examined birds were influenza A positive. From hundred and sixty four rRT-PCR positive samples a total of 45 influenza A viruses of different subtypes were isolated. The overall isolation rate was 6% (45/781). In our study many different influenza A virus subtypes were found to circulate at the same time, in the same bird species at the single location in the Northern Europe. This finding most likely indicates the existence of a large reservoir of different influenza A viruses in mallards population in Northern Europe. Eleven haemagglutinin- and nine different neuraminidase subtypes in twelve combinations have been isolated from apparently healthy mallards in the same geographical location (Figure 2). Mixing of migratory mallards at the single location may be the reason for the high level of virus variation. The most frequently identified subtypes in mallard populations in Northern Europe during autumn migration in 2005 were H3N8 (24%) and H4N6 (18%), similarly to the rates previously reported from North America and Europe [29,30]. Sequence analysis of the HA genes of the H5 and H7 influenza A viruses isolated in this study showed that the haemagglutinin cleavage site lacked the basic amino acids residues (data not shown), which indicating low pathogenicity of these viruses [31]. No highly pathogenic H5N1 viruses were isolated from mallards included in this study. This is important regarding the ongoing debate on the possible spread of HPAI H5N1 viruses by apparently healthy migratory birds and the time line of events characterising the first arrival of the HPH5N1 viruses in Western Europe and Baltic Sea area in winter 2005–2006 [32].

### Phylogenetic analysis

We analysed the *NS *gene sequences of the 45 influenza A viruses isolated from mallards in Northern Europe separately and together with selected number of isolates, reported between year 2000 to 2007, and previously published in the GenBank [33].

Analysis of phylogenetic relationships among the *NS *genes reported in this study clearly shows that two distinct gene pools, corresponding to both *NS *allele A and B [34], were present at the same time in the same geographic location in the mallards populations in Northern Europe. Out of 45 isolated viruses 39 (87%) belong to allele A, while six (13%) to allele B. Allele B viruses appear to be less common in natural host species than allele A, comprising only about 13% of the isolates sequenced in this study. The prevalence rates of allele B viruses in North American mallards are much higher than what we have seen in mallards in Northern Europe (30% in North America versus 13% in Northern Europe)[35]. In Asia the figure is 15 per cent, including all viruses of avian origin. Thus, the overall picture clearly shows that the majority of the viruses belong to allele A in birds.

The differences in function, if any, between allele A and allele B have not been defined, but it appears that allele B viruses are more distinct from mammalian origin viruses. All viruses from mammalian species belong to allele A, with only two exceptions, one previously reported equine origin virus (A/equine/Jilin/1/1989/H3N8) and as shown here, one swine origin virus (A/Swine/Saskatchewan/18789/2002/H1N1). However, both these viruses are believed to be a direct transmission from avian species [36,37]. Studies that have placed *NS *allele B gene into mammalian origin viruses have attenuated these viruses in mice [38]. This indicates that NS1 from allele B, cannot easily be adapted to mammalian species. Thus, it would be very interesting to be able to pinpoint possible differences in function between NS1 from allele A and B.

**Figure 3:**
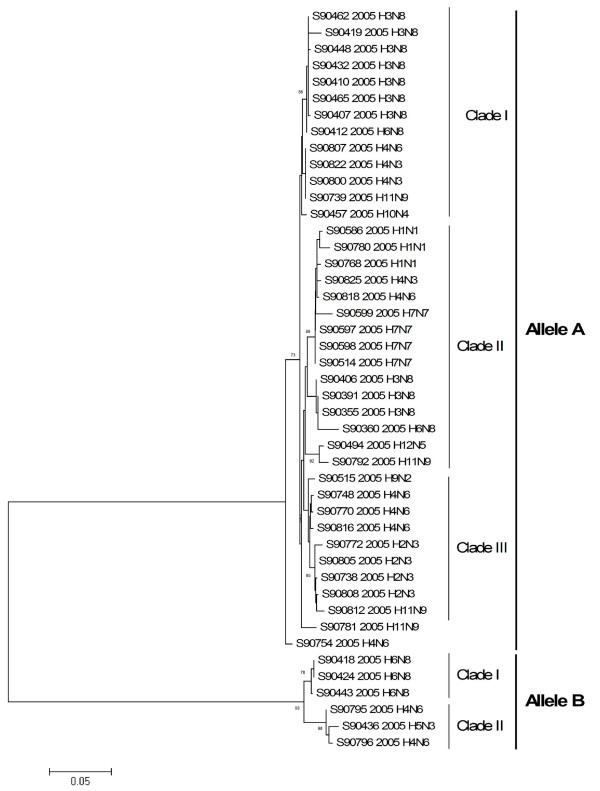
Phylogenetic relationship of NS1 genes of 45 influenza A viruses isolated from mallards in Northern Europe in 2005. The protein coding region tree was generated by neighbour-joining analysis with Tamura-Nei γ-model, using MEGA 4.0. Numbers below key nodes indicate the percentage of bootstrap values of 2000 replicates.

Phylogenetic analysis revealed three separate clades and multiple sub clades among isolates in allele A and two separate clades in allele B (Figure 3). Viruses in allele A were separated into three clades. Clade I consist of thirteen isolates divided into two sub clades. Clade II is encompassing fourteen isolates, divided into three subclades. Finally, twelve isolates formed clade III.

**Figure 4:**
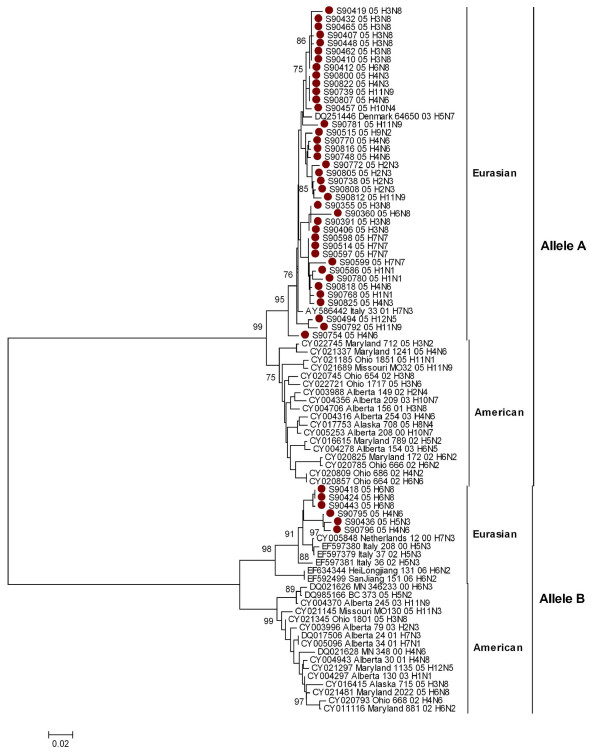
Phylogenetic relationship of NS1 genes of 45 influenza A viruses isolated from mallards in Northern Europe in 2005 compared with selected number of mallards isolates, reported between year 2000 to 2007, and previously published in the GenBank. The protein coding region tree was generated by neighbour-joining analysis with Tamura-Nei γ-model, using MEGA 4.0. Numbers below key nodes indicate the percentage of bootstrap values of 2000 replicates. Swedish isolates are indicated by red dot.

When co-analyzed with other viruses isolated from mallards the isolates grouped separately by Eurasian and American lineages in both alleles, without any geographical assortment of the mallard origin isolates (Figure 4).

**Figure 5:**
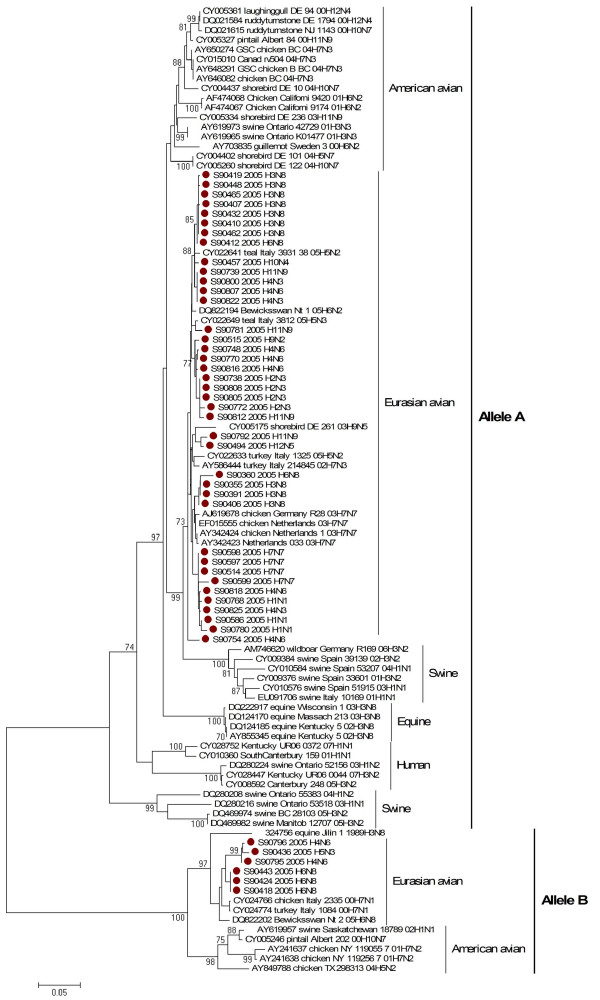
Phylogenetic relationship of NS1 genes of 45 influenza A viruses isolated i from mallards in Northern Europe in 2005 in comparison with virus genes from shorebirds, poultry and mammalian origin isolates, reported between year 2000 to 2007, and previously published in the GenBank. The protein coding region tree was generated by neighbour-joining analysis with Tamura-Nei γ-model, using MEGA 4.0. Numbers below key nodes indicate the percentage of bootstrap values of 2000 replicates. Swedish isolates are indicated by red dot.

Unlike pattern observed among mallard viruses, isolates from shorebirds shown some intercontinental exchange of genes (Figure 5). It has been shown by Wallensten and co-authors (2005) that *NS *gene segment of influenza A virus (A/Guillemot/Sweden/3/00/H6N2) isolated from Guillemot (*Uria aalge*) on Boden Island in the northern Baltic Sea belongs to American lineage of influenza A viruses [39]. Alternatively, as shown here, one *NS *allele A gene from A/shorebird/DE/261/03/H9N5 [40] fell into same clade with genes from Eurasian avian viruses (Figure 5).

The phylogenetic assortment appears to be more common among North American isolates, i.e. two swine origin isolates, A/swine/Ontario/42729/01/H3N3 and A/swine/Ontario/K01477/01/H3N3, grouped together with American avian origin viruses in allele A (Figure 5), however, limited sequence data is available from Eurasian origin viruses which make further conclusions difficult.

The viruses detected in poultry and in wild birds, grouped closely to each other in both alleles. The close relationship of the HPAI H7N7 isolates detected in 2003 in the Netherlands [41] and the LPAI isolate of the same subtype from apparently healthy mallards in Northern Europe in 2005 poses an important puzzle in the epidemiology of these viruses. This may indicate that viruses of the H7N7 subtype are currently circulating in the European Mallard bird population and these viruses still can constitute a threat to domestic poultry and public health.

### Molecular characterization

To further investigate the evolutionary stasis of the *NS *gene, we analyzed the nucleotide and protein sequences of NS1 and NEP of isolated viruses. Each of the *NS *genes consisted of 890 nucleotides; there were no deletions or insertions. Nucleotide sequence identities of *NS *gene within alleles were 95–100% and 97–100%, respectively; however, the two alleles were, at most, 72% similar (Table 1). In allele A viruses the largest divergence (5%) in nucleotide sequences was found between A/Mallard/Sweden/S90360/2005/H6N8 and A/Mallard/Sweden/S90419/2005/H3N8.

**Table 1:** Sequence similarity of the NS gene products among influenza A viruses isolated in Northern European mallards.

	NS1 % similarity		NEP % similarity	
	
Comparsion	Aminoacids	Nucleotide	Aminoacids	Nucleotide
Within allele A	95–100%	95–100%	88–100%	93–100%
Within allele B	98–100%	97–100%	95–100%	90–100%
Between allele A and B	68–72%	67–70%	76–83%	71–77%

The nucleotide sequence of the NEP consists of 363 nucleotides encoded from a spliced mRNA. The potential splice donor and acceptor sites were conserved in the entire *NS *gene examined in this report (data not shown). Within the allele A and B, the NEP showed a nucleotide similarity of at least 85 and 90%, respectively, between the two alleles, the nucleotide similarity was 77% at most.

The nucleotide sequences of isolated viruses were compared for similarity. The A/tern/South Africa/1961/H5N3 and A/redhead duck/ALB/74/1977/H4N6[40] which represent the earliest isolates from wild birds reservoir were used as a baseline for respectively allele A and allele B viruses. Thirty-one nucleotide substitutions were found among clade I viruses in allele A compared to reference strain. Of these, twenty-six were transitions; 14 were pyrimidine and 12 were purine transitions and five substitutions were results of transversion. Five of these substitutions resulted in amino acid changes in NS1 protein. Analysis of the sequence variations demonstrated that nucleotide changes are not uniformly distributed across the gene with a few relatively variable site identified at the N-terminus of the effector domain. In clade II viruses, thirty-four substitutions were observed compared to A/tern/South Africa/1961/H5N3. Of these, thirty-one were result of transitions (17 T or C substitution and 14 A or G substitutions). Four of these substitutions resulted in amino acid changes in NS1 protein. Thirty-two nucleotide substitutions were found in viruses belong to clade III. Six amino acid changes in NS 1 protein were results of these substitutions, two located in RNA binding domain and 4 in effector domain of the NS1 protein. Sixty-three nucleotide substitutions were found among clade I viruses in allele B compared to reference strain. Fourty-one of these were transitions; 23 of these were pyrimidine and 18 were purine transitions. Only 3 of these substitutions resulted in amino acid changes in NS1 protein. In the genome of clade II viruses 58 substitutions were observed compared to A/redhead duck/ALB/74/1977/H4N6. Thirty-nine of these were results of transitions (20 T or C and 19 A or G substitutions). Three of these substitutions resulted in amino acid changes in NS1 protein.

Two hundred and four (30%) nucleotide substitutions were found among viruses in allele B compared to A/tern/South Africa/1961/H5N3. Of these, 91 were result of transitions. These substitutions were resulted to 70 amino acid differences between the allele B viruses and A/tern/South Africa/1961/H5N3. These results are similar to those previously reported by Suarez and Perdue [42].

**Figure 6:**
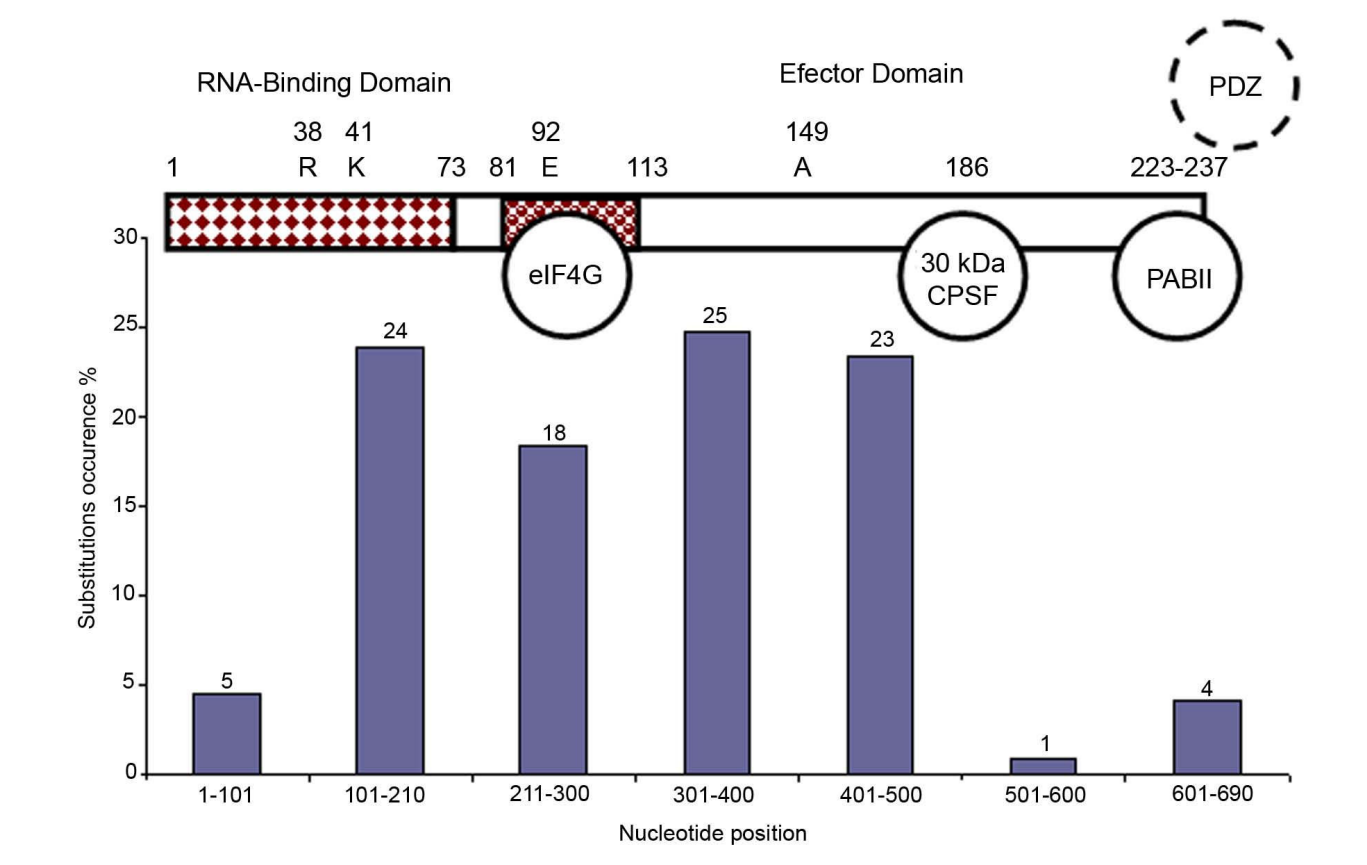
Frequency of substitution at the nucleotide position of NS1 gene among studied viruses.

Analysis of the sequence variations demonstrated that nucleotide changes are almost uniformly distributed across the whole gene with only one relatively conserved site at the 3' end of the nucleotide sequence (Figure 6). A comparison of nucleotide sequences of isolated viruses revealed a substantial number of silent mutations, which results in high degree of homology in protein sequences. The degree of variation within the alleles is very low. Allele A viruses displays a maximum of 5% amino acid divergence while allele B viruses display only 2% amino acid divergence.

The length of NS1 protein in some influenza A viruses isolated from poultry and mammalian hosts has been shown to vary, but the NS1 protein of all the isolates of either subtypes presented in this study consist of 230 amino acid residues without any insertion or deletions. In its natural host, the *NS *gene evolves slowly, but when introduced into a new host the evolution goes rather fast which can results in deletions, insertions and truncations of NS1 [43,44].

Several studies have identified important amino acid residues for the function of NS1 protein in the infected cells [7,10,16-18]. Our knowledge about the existence of these motifs in the *NS *gene pool of influenza A viruses in their natural reservoirs is insufficient. To further evaluate the existence of these specific motifs in our data set we aligned additional 4073 amino acid sequences, available at the GenBank, together with the data generated in this study. Two major functional domains have been suggested on NS1 protein, the N-terminal RNA-binding domain (residues 1–73) and the C-terminal effector domain (residues 73–237) [3]. The arginine at position 38 and the Lysine at position 41 contribute to both dsRNA binding activity and interferon antagonist activity of the NS1 protein [10]. The NS1 gene of all studied isolates includes R38 and K41. We found only two avian influenza viruses: A/Pintail/Alberta/1979/H4N6 and A/Chukkar/MN/1998/H5N2 among 4073 studied viruses that contained substitution at the position 38; R38A and R38K respectively. The substitution at amino acid position 41 appear more frequently in human isolates of subtypes H1N2 and H3N2 and swine isolates of subtypes H3N2, while the K41 seem to be much more conservative in avian and equine isolates. The absolute majority of human H1N2 and H3N2 viruses contain substitution K41R. This substitution has also been seen in A/Swine/Ontario/52156/2003/H1N2 that phylogenetic grouped with human influenza A viruses.

The amino acid Glu92 in the NS1 protein observed in H5N1/97 influenza viruses is implicated in their ability to modulate the cytokine response and has been associated with the high virulence of these viruses in pigs [45]. At the GenBank database only 26 H5N1 viruses contains Glu92, mostly isolated in Hong-Kong in 1997. Among avian isolates six H6N1 and several H9N2 viruses contains Glu92. Interestingly one swine isolate; A/swine/United Kingdom/119404/91/H3N2, also contain Glu92 in the NS1 protein. No viruses sequenced in this study contained glutamic acid at position 92 of the NS1 protein. Overall, the substitution of Glu92 is extremely rare, and the importance for the virulence in other species than pigs is unclear.

It has been suggested that the amino acid at the position 149 of NS1 protein of HPAI-H5N1 affect the ability of the virus to antagonize the induction of IFN α/β in chicken embryo fibroblasts [46]. All Swedish isolates sequenced in this study possessed the amino acid Ala149 in their NS1 protein and have this proposed virulence hallmark of NS1.

The NS1 protein interaction with cleavage and polyadenylation specificity factor (CPSF) inhibits 3'-end processing of cellular pre-mRNA [16-18]. This function mediated by two distinct domains; one around residue 186 [18] and the other one around residue 103 and 106 [19]. All isolates sequenced in this study possessed the amino acid Glu186, Phe103 and Met106 in their NS1 protein.

It was proposed earlier by Obenauer and colleagues (2006) that NS1 have a PDZ binding motif at the very end of the protein. PDZ domains are protein-interacting domains present once or multiple times within certain proteins and these domains are involved in the cell signalling, assembly of large protein complexes or intracellular trafficking. They also showed that there were typical human, avian, equine and swine motifs. The most commonly seen avian motif ESEV were shown to bind to several PDZ domains in human proteins, while the most common human motif RSKV bound very few [40]. All the viruses isolated from mallards in Northern Europe possessed the typical avian ESEV amino acid sequence at the C-terminal end of the NS1 protein. However, viruses from Asia have slightly other versions, like EPEV and GPEV. The EPEV motif appears in both avian as well as swine, human and equine viruses [39]. It is therefore possible that this motif of NS1 is important for the adaptation of influenza into a new host. The exact functional relevance of this remains unclear at the moment.

The NEP of the studied isolates consists of 121 amino acids. It has been suggested that tryptophan at position 78 is involved in NEP-M1 interaction that mediates the nuclear export of viral ribonucleoprotein complexes [23]. All Swedish isolates sequenced in this study possessed the amino acid TRP78 in their NEP. Hayman and co-workers suggested that two differences in the sequence of the NEP, at position 14 and 70, are particularly important for the attenuation of replication of the avian influenza viruses in human [47]. All the viruses studied here contain avian methionine/glutamine at position 14 and avian serine at position 70.

## Conclusion

Our surveillance study indicates existence of a large reservoir of different influenza A viruses in mallards population in Northern Europe. Twenty four per cent of examined birds were influenza A positive. Eleven haemagglutinin- and nine different neuraminidase subtypes in twelve combinations have been isolated, including the low pathogenic H5N3 and H7N7.

Finally, to our knowledge, this is the first study providing a comprehensive analysis of *NS *gene of avian influenza in its natural reservoir in Europe. Our findings improve the present understanding of *NS *gene pool of avian influenza viruses and should help in understanding of gene function in the natural host, mallards, as well as in other hosts, like domestic avian species. Particularly interesting is the fact that two distinct gene pools, corresponding to both *NS *allele A and B, were present in the mallard populations in Northern Europe. Allele B viruses appear to be less common in natural host species than allele A, comprising only about 13% of the isolates sequenced in this study. Despite the high level of subtype variation among studied viruses the nucleotide sequences of *NS *gene of these viruses showed a substantial number of silent mutations, which results in high degree of homology in protein sequences.

## Methods

### Field sampling of live wild birds

Samples were collected at the Ottenby bird observatory from seven hundred and eighty one mallards (*Anas platyrhynchos*) in the frame of a surveillance program, organized by the Swedish Board of Agriculture. The Ottenby bird observatory is situated on a major European flyway, in Baltic island of Öland in southeast coast of Sweden (Figure 1). Birds were caught from October until the autumn migrations were ended in late December. After banding and collection of biometrical data, two cloacal swabs or fresh dropping samples were taken from each bird using cotton swabs and stored in transport media at -70°C until processed. Transport media consisted of Hanks balanced salt solution supplemented with 10% glycerol, 200 U/ml penicillin, 200 μg/ml streptomycin, 100 U/ml polymyxin B sulphate, 250 μg/ml gentamicin, and 50 U/ml nystatin (all from ICN, Zoetermeer, the Netherlands). All samples were strictly handled in a government-certified biosafety level 3+ (BSL-3+) facilities by highly trained staff. Collected samples were screened for the presence of influenza A viruses by real-time reverse transcriptase polymerase chain reaction (rRT-PCR) for the matrix protein gene [48], all positive cases were further analysed by conventional reverse transcriptase-PCR (RT-PCR) for detection of H5 and H7 viruses, including virus pathotyping by amplicon sequencing of the identified H5 and H7 viruses [49]. All PCR assays were performed according to the recommendations from the Community Reference Laboratory (CRL; VLA Addlestone).

### Virus isolation and characterisation

Virus isolation was performed in a BSL3+ laboratory at the National Veterinary Institute (SVA) in Sweden. Samples that were identified as influenza A virus positive by matrix rRT-PCR were thawed, mixed with an equal volume of phosphate buffered saline containing antibiotics (penicillin 2000 U/ml, streptomycin 2 mg/ml and gentamicin 50 μg/ml), incubated for 20 minutes in room temperature, and centrifuged at 1,500 × g for 15 min. The supernatant (0.2 ml/egg) was inoculated into the allantoic cavity of four 9-days old specific pathogen free (SPF) embryonated hens' eggs as described in European Union Council Directive 92/40/EEC [50]. Embryonic death within the first 24 hours of incubation was considered as non-specific and these eggs were discarded. After incubation at 37°C for 3 days the allantoic fluid was harvested and tested by haemagglutination (HA) assay as describe in European Union Council Directive 92/40/EEC. In the cases where no influenza A virus was detected on the initial virus isolation attempt, the allantoic fluid was passaged twice in embryonated hens eggs. The number of virus passages in embryonated eggs was limited to the maximum two, to limit laboratory manipulation. A sample was considered negative when the second passage HA test was negative. The subtypes of the virus isolates were determined by conventional haemagglutination inhibition (HI) test, as describe in European Union Council Directive 92/40/EEC and the neuramidinase inhibition (NI) test [51].

### RNA extraction and PCR with NS1 gene specific primers

RNA was extracted in a BSL-3+ laboratory, using Trizol reagent (Invitrogen Corp., Carlsbad, CA) according to the manufacturer's instructions. The RNA was converted to full-length cDNA using reverse transcriptase. The RT mix comprised 2.5 μl of DMPC water, 5 μl of 5× First Strand buffer (Invitrogen), 0.5 μl of 10 mM dNTP mix (Amersham Biosciences), 2 μl of 50 mM random primers (pdN6), 32 U of RNAguard (Amersham Biosciences), 200 U of MMLV reverse transcriptase (Invitrogen) and 5 μl RNA solution in total volume of 25 μl. The reactions were incubated at 42°C for 90 min followed by inactivation of the enzyme at 95°C for 5 min.

PCR amplification with NS gene specific primers (Fw primer: 5' AGC AAA AGC AGG GTG ACA AAG 3', Rev primer 5' AGT AGA AAC AAG GGT GTT TTT TAT 3') was performed to amplify the product containing the full length NS gene. Twenty-five microliter PCR-mix contained 1× Platinum Taq buffer (Invitrogen), 200 μM dNTP, 2.5 mM MgCl_2_, 240 nM each of Fw primer and Rw primer, 1 U Platinum Taq DNA Polymerase (Invitrogen) and 3 μl cDNA. Reactions were placed in a thermal cycler at 95°C for 2 min, then cycled 35 times between 95°C 20 sec, annealing at 58°C for 60 sec and elongation at 72°C for 90 sec and were finally kept at 8°C until later use.

The PCR products were treated with shrimp alkaline phosphatase-exonuclease I (ExoSapI) (U.S Biologicals, Swampscott, MA, USA) (5 μl ExoSapI per reaction, 30 min. at 37°C followed by 10 min. at 95°C) and utilized for sequencing directly.

### NS1 sequences obtained from GenBank

The NS gene was analysed both with selected number of mallards isolates and in comparison with virus genes from poultry and mammalian origin isolates.

The NS1 gene sequences of 100 additional influenza A viruses, reported between year 2000 to 2007, obtained from GenBank were used in phylogenetic studies [33].

### Phylogenetic and sequence analysis

Sequences of the purified PCR products were determined using gene specific primers and BigDye Terminator version 3.1 chemistry (Applied Biosystems, Foster City, CA), according to the manufacturer's instructions. Reactions were run on a 3100 DNA analyzer (Applied Biosystems). Sequencing was performed at least twice in each direction. After sequencing, assembly of sequences, removal of low-quality sequence data, nucleotide sequence translation into protein sequence, additional multiple sequence alignments and processing were performed with the Bioedit software version 7.0.4.1[52] with an engine based on the Custal W algorithm [53]. Blast homology searches  were used to retrieve the top fifty homologous sequences for the sequenced gene from the GenBank database. The phylogenetic analysis, based on complete gene nucleotide sequences were conducted using Molecular Evolutionary Genetics Analysis (*MEGA*, version 4.0) software [54] using neighbour-joining tree inference analysis with the Tamura-Nei γ-model, with 2000 bootstrap replications to assign confidence levels to branches.

### Nucleotide sequence accession numbers

The nucleotide sequence data obtained in this study has been submitted to the GenBank database and is available under accession numbers; EU518715–EU518759 (Table 2).

**Table 2:** Influenza A virus isolates collected from Mallards in Northern Europe in 2005.

**Viruses**	**Accession**	**Allele**
A/Mallard/Sweden/S90355/2005/H3N8	EU518715	Allele A
A/Mallard/Sweden/S90360/2005/H6N8	EU518716	Allele A
A/Mallard/Sweden/S90391/2005/H3N8	EU518717	Allele A
A/Mallard/Sweden/S90406/2005/H3N8	EU518718	Allele A
A/Mallard/Sweden/S90407/2005/H3N8	EU518719	Allele A
A/Mallard/Sweden/S90410/2005/H3N8	EU518720	Allele A
A/Mallard/Sweden/S90412/2005/H6N8	EU518721	Allele A
A/Mallard/Sweden/S90418/2005/H6N8	EU518722	Allele B
A/Mallard/Sweden/S90419/2005/H3N8	EU518723	Allele A
A/Mallard/Sweden/S90424/2005/H3N8	EU518724	Allele B
A/Mallard/Sweden/S90432/2005/H3N8	EU518725	Allele A
A/Mallard/Sweden/S90436/2005/H5N3	EU518726	Allele B
A/Mallard/Sweden/S90443/2005/H6N8	EU518727	Allele B
A/Mallard/Sweden/S90448/2005/H3N8	EU518728	Allele A
A/Mallard/Sweden/S90457/2005/H10N4	EU518729	Allele A
A/Mallard/Sweden/S90462/2005/H3N8	EU518730	Allele A
A/Mallard/Sweden/S90465/2005/H3N8	EU518731	Allele A
A/Mallard/Sweden/S90494/2005/H12N5	EU518732	Allele A
A/Mallard/Sweden/S90514/2005/H7N7	EU518733	Allele A
A/Mallard/Sweden/S90515/2005/H9N2	EU518734	Allele A
A/Mallard/Sweden/S90586/2005/H1N1	EU518735	Allele A
A/Mallard/Sweden/S90597/2005/H7N7	EU518736	Allele A
A/Mallard/Sweden/S90598/2005/H7N7	EU518737	Allele A
A/Mallard/Sweden/S90599/2005/H7N7	EU518738	Allele A
A/Mallard/Sweden/S90738/2005/H2N3	EU518739	Allele A
A/Mallard/Sweden/S90739/2005/H11N9	EU518740	Allele A
A/Mallard/Sweden/S90748/2005/H4N6	EU518741	Allele A
A/Mallard/Sweden/S90754/2005/H4N6	EU518742	Allele A
A/Mallard/Sweden/S90768/2005/H1N1	EU518743	Allele A
A/Mallard/Sweden/S90770/2005/H4N6	EU518744	Allele A
A/Mallard/Sweden/S90772/2005/H2N3	EU518745	Allele A
A/Mallard/Sweden/S90780/2005/H1N1	EU518746	Allele A
A/Mallard/Sweden/S90781/2005/H11N9	EU518747	Allele A
A/Mallard/Sweden/S90792/2005/H11N9	EU518748	Allele A
A/Mallard/Sweden/S90795/2005/H4N6	EU518749	Allele B
A/Mallard/Sweden/S90796/2005/H4N6	EU518750	Allele B
A/Mallard/Sweden/S90800/2005/H4N3	EU518751	Allele A
A/Mallard/Sweden/S90805/2005/H2N3	EU518752	Allele A
A/Mallard/Sweden/S90807/2005/H4N6	EU518753	Allele A
A/Mallard/Sweden/S90808/2005/H2N3	EU518754	Allele A
A/Mallard/Sweden/S90812/2005/H11N9	EU518755	Allele A
A/Mallard/Sweden/S90816/2005/H4N6	EU518756	Allele A
A/Mallard/Sweden/S90818/2005/H4N6	EU518757	Allele A
A/Mallard/Sweden/S90822/2005/H4N3	EU518758	Allele A
A/Mallard/Sweden/S90825/2005/H4N3	EU518759	Allele A

## Competing interests

The authors declare that they have no competing interests.

## Authors' contributions

SZ conceived and designed the study, organized protocol developments, carried out PCR and sequencing reactions, performed sequence analyses, alignments, phylogenies, interpretation of data, carried out identification of viruses and wrote the manuscript. PG took part in development of amplification protocols, contributed to and revised the manuscript. AE, provided nucleotide sequences and core data, contributed to the interpretation of the findings and revised the manuscript. UB propagated the viruses, contributed to the interpretation of the findings and revised the manuscript. ME, provided nucleotide sequences and core data, contributed to the interpretation of the findings and revised the manuscript. PT, GC and SB contributed to conception, interpretation of data, and revised the manuscript. JW and BO developed the sampling design and directed the collection of samples and revised the manuscript. MB additionally contributed to the study design and revised the manuscript. All authors' have read and approved the final manuscript.
